# Establishing Mycophenolate Therapeutic Drug Monitoring Targets in Childhood Nephrotic Syndrome

**DOI:** 10.1016/j.ekir.2026.107131

**Published:** 2026-10-06

**Authors:** Magdalena Riedl Khursigara, Cal H. Robinson, Erin Chung, Winnie Seto, Diana Hamdan, Junhee Hong, Valentina Bruno, Nowrin Aman, Tonny H.M. Banh, Judy Truong, Josefina Brooke, Vaneet Dhillon, Mackenzie Garner, Christoph Licht, Ashlene McKay, Damien Noone, Rachel Pearl, Seetha Radhakrishnan, Keisha Rasool, Nithiakishna Selvathesan, Kristen Vanos, Jovanka Vasilevska-Ristovska, Rulan S. Parekh, James Tjon, Chia Wei Teoh

**Affiliations:** 1 Division of Nephrology, The Hospital for Sick Children, Toronto, Ontario, Canada; 2 Kidney Disease Initiative, Broad Institute, Cambridge, Massachusetts, USA; 3 Child Health Evaluative Sciences, Research Institute, The Hospital for Sick Children, Toronto, Ontario, Canada; 4 Institute of Health Policy, Management and Evaluation, University of Toronto, Toronto, Ontario, Canada; 5 Department of Pharmacy, The Hospital for Sick Children, Toronto, Ontario, Canada; 6 Leslie Dan Faculty of Pharmacy, University of Toronto, Toronto, Ontario, Canada; 7 College of Osteopathic Medicine, Kansas City University, Joplin, Missouri, USA; 8 Department of Pediatrics, Faculty of Medicine, University of Toronto, Toronto, Ontario, Canada; 9 Department of Medicine, Women’s College Hospital, Toronto, Ontario, Canada; 10 Division of Nephrology, Stollery Children’s Hospital, Edmonton, Alberta, Canada; 11 Department of Pediatrics, William Osler Health Systems, Ontario, Canada

**Keywords:** area under the curve, mycophenolic acid, nephrotic syndrome, therapeutic drug monitoring

## Abstract

**Introduction:**

Mycophenolic acid (MPA) is a commonly used steroid-sparing immunosuppressant in nephrotic syndrome. Therapeutic drug monitoring (TDM) based on area under the concentration-time curve (AUC) target ranges is not well-established. We aimed to propose exposure TDM targets for MPA in children with nephrotic syndrome when used concomitantly with rituximab.

**Methods:**

We performed a retrospective analysis of children with nephrotic syndrome treated with mycophenolate mofetil (MMF) or enteric-coated mycophenolate sodium (EC-MPS). Exposures included MPA-AUC_0-12h_, MPA trough concentrations, and MMF equivalent dose. Clinical outcomes included relapse rate, time-to-relapse, and adverse effects. We determined a favorable MPA-AUC_0-12h_ range, based on time-to-relapse and adverse effects. Poisson regression models with natural splines and receiver operating characteristic curves were used.

**Results:**

A total of 131 MPA-AUC_0-12h_ measurements were performed among 67 children with frequently relapsing nephrotic syndrome (FRNS). MPA-AUC_0-12h_, but not trough concentration or MMF equivalent dose, was strongly correlated with relapse rate and time-to-relapse. A 10 mg h/l increase of MPA-AUC_0-12h_ was associated with fewer relapses (incidence rate ratio [IRR], 0.70; 95% confidence interval [CI], 0.59–0.82) and longer time-to-relapse (hazard ratio [HR], 0.77; 95% CI: 0.64–0.92). For relapse rate, the best model fit was achieved using 2 knots at MPA-AUC_0-12h_ of 41 and 57 mg h/l. Higher MPA-AUC_0-12h_, especially ≥ 60 mg h/l, was associated with increased risk of neutropenia (odds ratio, 1.18 per 10 mg h/l increase; 95% CI, 1.02–1.36).

**Conclusion:**

Our findings suggest an initial target MPA-AUC_0-12h_ between 40 to 60 mg h/l provided a favorable balance between relapse prevention and adverse effects. Further increases of MPA-AUC_0-12h_ > 60 mg h/l may provide further relapse risk reduction, but should be carefully weighed against neutropenia risk.

## Graphical abstract

**Figure:**
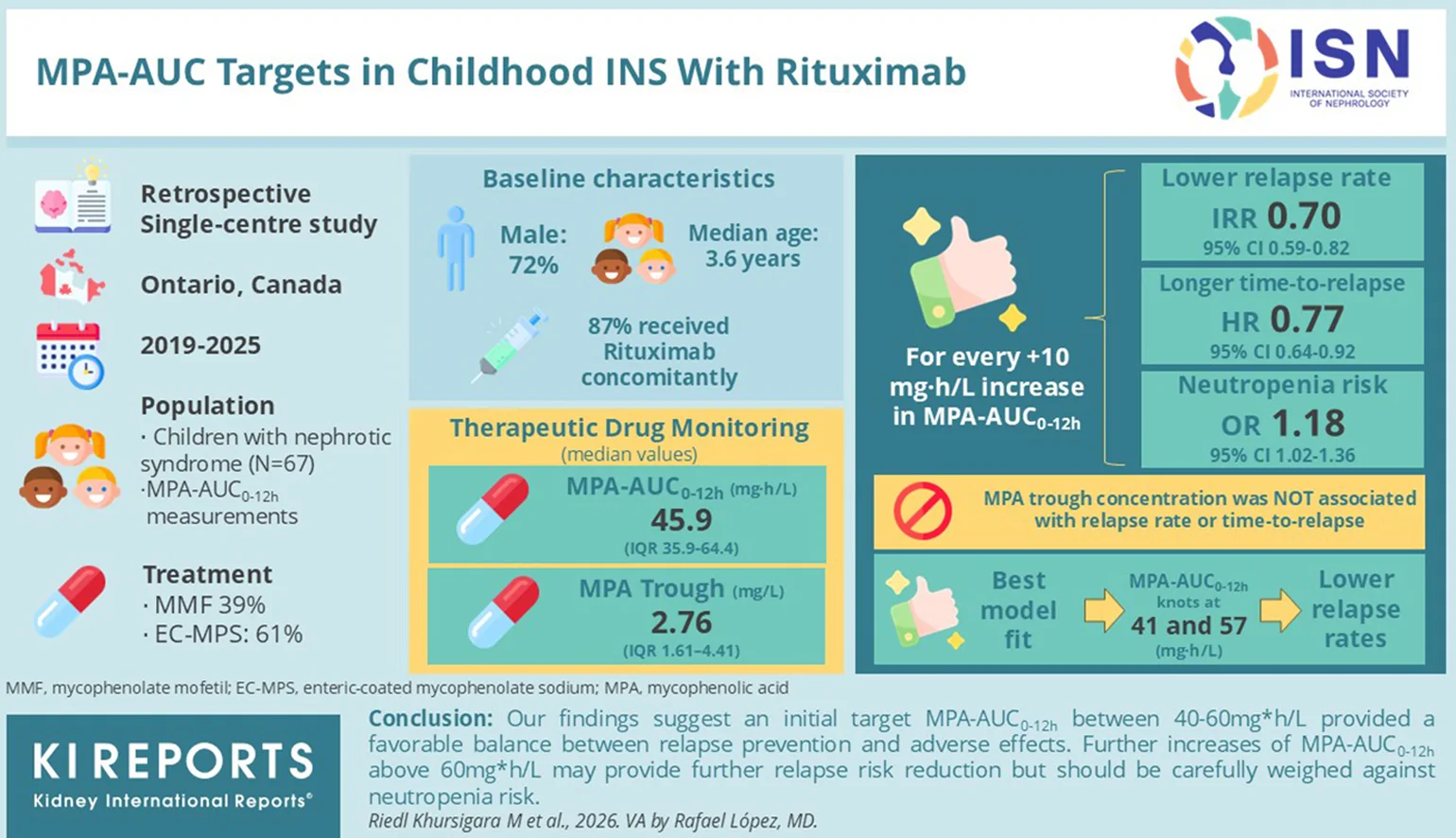

Nephrotic syndrome is the most common glomerular disease affecting children.^1^ Its etiology is not entirely understood; however, immune dysregulation is an important cornerstone of disease pathology. Two-thirds of children relapse and 31% follow a frequently relapsing course(i.e., FRNS).^2^ Patients with FRNS are at risk for steroid toxicity, such as obesity, cataracts, hypertension, and growth suppression, due to prolonged use of steroids.^2^, ^3^, ^4^, ^5^ Therefore, steroid-sparing agents, such as cyclophosphamide, calcineurin-inhibitors (cyclosporine or tacrolimus), antimetabolite MPA, or anti-CD20 monoclonal antibody (rituximab) are used to maintain sustained remission and prevent steroid toxicity.^6^, ^7^ There is currently limited evidence on which second-line agents are superior to others.^8^, ^9^, ^10^, ^11^ In nephrotic syndrome, use of steroid-sparing agents varies regionally; MPA is either used as a sole second-line agent^10^, ^12^ or as a concomitant agent with an anti-CD20 monoclonal antibody.^13^ MPA monotherapy reduces relapses and cumulative steroid dose,^14^ but may be considered less effective when compared with calcineurin inhibitors.^8^ Combining rituximab with MPA has been shown to prolong steroid-free remission.^13^ However, few studies have identified the dose or drug level associated with improvements in clinical outcomes, or the optimal method for measuring it.

MPA is a nonnephrotoxic immunosuppressant with an inhibitory effect on inosine monophosphate dehydrogenase, a key enzyme of T- and B-cell proliferation.^15^ MPA can be given as MMF, a prodrug that is rapidly hydrolyzed into its active form upon absorption, or as EC-MPS, a delayed-release formulation absorbed directly in the small intestine. TDM includes MPA trough concentrations and/or MPA-AUC_0-12h_ monitoring.^10^, ^16^, ^17^, ^18^, ^19^, ^20^ Target MPA exposure, measured by MPA-AUC_0-12h_, varies dramatically between studies. Most studies suggest a lower cut-off ranging from 30 to 50 mg h/l,^10^, ^16^, ^17^, ^18^ with only a few studies reporting the effect on relapse rates. Higher MPA-AUC_0-12h_ targets are associated with improved relapse control.^7^, ^10^ No study has ever established an upper limit associated with adverse effects, including hematologic abnormalities such as anemia, leukopenia, neutropenia, and/or thrombocytopenia.^16^ In addition, the limited use of established target ranges has made it difficult to interpret trial outcomes.^21^ Further, MPA exposure in patients receiving concomitant treatment of MPA and rituximab remains largely unexplored.

The study aims were as follows: (i) to compare weight-based MPA dosing with TDM (trough concentrations and MPA-AUC_0-12h_) in terms of their association with relapse rates and time-to-relapse, and (ii) to propose exposure MPA-AUC_0-12h_ targets in children with FRNS, with particular focus on those receiving MPA concomitantly with rituximab, by assessing relapse rates and laboratory-related adverse effects.

## Methods

### Study Design

We performed a retrospective analysis of all MPA-AUC_0-12h_ measured between November 2019 and June 2025 in children with nephrotic syndrome at a single center. In addition, linked clinical follow-up data from the Insight into Nephrotic Syndrome: Investigating Genes, Health, and Therapeutics (INSIGHT) study were included.^22^ INSIGHT is a prospective, longitudinal cohort of childhood nephrotic syndrome, which has enrolled children from the Greater Toronto and Hamilton Area diagnosed with nephrotic syndrome since 1996, with prospective follow-up since 2011.^22^ Research ethics approval was obtained from The Hospital for Sick Children Research Ethics Board (approval number: 1000021384) and the study was conducted in accordance with the ethical standards of the Declaration of Helsinki. This manuscript was reported in accordance with the Strengthening the Reporting of Observational Studies in Epidemiology (STROBE) guidelines (Supplementary Appendix S1).^23^

### Study Population

We included all children (aged 1–18 years) diagnosed with nephrotic syndrome who were treated with MMF (capsule, tablet, or oral suspension) or EC-MPS (tablet) and underwent MPA TDM between November 2019 and June 2025. At our institution, a quaternary pediatric hospital in Ontario, Canada, patients with FRNS typically receive a course of oral cyclophosphamide or tacrolimus as initial steroid-sparing medications.^9^ If relapses persist or if there are concerns of toxicity, patients are then treated with rituximab followed by MPA as concomitant maintenance immunosuppression, with the intention of avoiding and/or delaying the need for repeated rituximab doses in order to minimize the risk from longer-term adverse effects such as hypogammaglobulinemia.^24^ The dose of rituximab is not routinely repeated based on CD19^+^ cell repopulation. The choice of MMF or EC-MPS formulation is determined by the treating physician, influenced by the patient’s ability to swallow tablets or capsules or liquid suspension (only MMF was available in a liquid suspension), and dependent on drug coverage in Ontario. Children were excluded if they were aged < 1 year, had genetic or secondary nephrotic syndrome, or were diagnosed with another form of glomerulonephritis. Nephrotic syndrome is defined using the standardized Kidney Disease Improving Global Outcomes criteria (i.e., the presence of proteinuria [urine protein-to-creatinine ratio ≥ 200 mg/mmol, or ≥ 3+ protein on urine dipstick], and either hypoalbuminemia [≤ 30 g/l], or edema).^25^, ^26^ In addition, steroid sensitivity and resistance were classified based on the Kidney Disease Improving Global Outcomes criteria.^25^, ^26^, ^27^ FRNS is defined as ≥ 2 relapses in the first 6 months or  ≥ 3 relapses in any 12 months. Steroid-dependent nephrotic syndrome was defined as 2 relapses within 162 days of diagnosis or 95 days of a previous relapse, which corresponds to 2 consecutive relapses during or within 14 days of discontinuing steroids (based on standardized local treatment protocols).

### Therapeutic Drug Monitoring of MPA

TDM of MPA was performed approximately 1 month after the target MMF/EC-MPS dose was achieved, and after any subsequent dose changes while the patient was in remission. Additional MPA measurements were often performed to further guide MMF/EC-MPS dose optimization in case of ongoing relapses or side effects. Serum MPA concentrations were determined using liquid chromatography tandem mass spectrometry at a local hospital-based clinical laboratory. A SCIEX 4000 QTRAP mass spectrometer with a coefficient of variation and/or accuracy of < 5% was used. The assay limit of quantification is < 0.14 mg/l. Three AUC calculations included MPA measurements below the limit of quantification, for which a fixed value of 0.07 mg/l was used. For patients receiving EC-MPS, 8 serum MPA concentrations were collected over 12 hours (0 h [predose], 1 h, 2 h, 4 h, 6 h, 8 h, 10 h, 12 h postdose), because limited sampling strategies have not been well-established for EC-MPS due to its pharmacokinetic variability.^28^, ^29^ The MPA-AUC_0-12h_ was then calculated using the conventional linear trapezoidal rule,^30^ using the following estimation equation for MPA-AUC = ([C_0_ + C_1_] / 2) × (t_1_−t_0_) + ([C_1_ + C_2_] / 2) × (t_2_ − t_1_) + ([C_4_ + C_2_] / 2) × (t_4_ − t_2_) + ([C_6_ + C_4_] / 2) × (t_6_ − t_4_) + ([C_8_ + C_6_] / 2) × (t_8_ − t_6_) + ([C_10_ + C_8_] / 2) × (t_10_ − t_8_) + ([C_12_ + C_10_] / 2) × (t_12_ − t_10_). For patients receiving MMF, a limited sampling strategy^31^ was used with measurements at 0 h (predose), 1 h, 2 h, and 6 h postdose to allow for the inclusion of a later peak to assess for enterohepatic recirculation of MPA.^32^, ^33^, ^34^ The following equation was used for the calculation of the MPA-AUC = 10.75 + 0.98C_1_ + 2.38C_2_ + 4.86C_6_^31^.

### Exposures

Our key exposures were MPA-AUC_0-12h_, MPA trough concentration, and MMF equivalent dose per body surface area and weight. For patients on EC-MPS, the MMF equivalent dose was calculated, for example, 720 mg of EC-MPS is equivalent to 1000 mg of MMF.^35^ The observation window was defined as the period from the start of the MMF/EC-MPS dose until the dose was either (i) changed, (ii) discontinued, or (iii) the last follow-up was recorded. MMF/EC-MPS was usually initiated at an MMF equivalent dose of 600 mg/m^2^/d, and titrated up to the maximally tolerable dose, aiming for a target dose of 1200 mg/m^2^/d within 4 weeks. Changes to the MMF/EC-MPS dose were made by the treating physician, considering time from last relapse, side effects observed by the physician or the family, and MPA-AUC_0-12h_.

### Outcomes

Our primary outcome was the relapse rate within each observation window. To propose exposure MPA-AUC_0-12h_ targets, we modelled the association between MPA-AUC_0-12h_ and nephrotic syndrome relapse rate and laboratory adverse effects. Relapses were defined as recurrence of nephrotic range proteinuria (i.e., 3+ urine dipstick protein for ≥ 3 consecutive days or urine protein-to-creatinine ratio > 200 mg/mmol).^26^ Relapses were primarily detected using home urine monitoring and often confirmed using laboratory urine testing. At our institution, families are educated to perform home urine testing several times per week and daily during intercurrent illnesses or if edema is present. Time-to-relapse within each observation window was evaluated as a secondary outcome. Laboratory-related adverse effects were defined using the following criteria: anemia: hemoglobin < 110 g/l, leukopenia: white blood cells < 4 × 10^9^/l, thrombocytopenia: platelets < 150 × 10^9^/l, neutropenia: absolute neutrophil count < 1.5 × 10^9^/l, transaminitis: alanine aminotransferase or aspartate aminotransferase > 2 × upper limit of normal for age or sex, and hypercholesterolemia: total cholesterol ≥ 5.2 mmol/l or low-density lipoprotein cholesterol ≥ 3.4 mmol/l. Laboratory tests were conducted monthly, including CD19^+^ cells for patients receiving concomitant rituximab.

### Statistical Analysis

All statistical analyses were performed using R Statistical Software (version 4.4.0, Vienna, Austria), and a *P*-value < 0.05 was considered significant. We evaluated baseline characteristics stratified among the overall population using descriptive statistics. Correlations between MPA-AUC_0-12h_, MPA trough concentration, and MMF equivalent dose (by weight and body surface area) were assessed using Pearson correlation coefficients (*r*), coefficients of determination (*R*^*2*^), and plots with linear regression lines.

We evaluated associations between MPA-AUC_0-12h_, MPA trough concentration, and MMF equivalent dose with nephrotic syndrome relapse rate using generalized estimating equations (GEE) with Poisson links to calculate unadjusted IRRs. GEE was used to account for data clustering, because multiple MPA dose observation windows per patient were included. In addition, we evaluated associations between MPA-AUC_0-12h_, MPA trough concentration, and MMF equivalent dose quartiles with relapse rate using GEE with Poisson links. Associations between MPA-AUC_0-12h_, MPA trough concentration, and MMF equivalent dose with time-to-relapse were evaluated using Cox proportional hazard models to calculate unadjusted HRs and plotted using the Kaplan-Meier method. Frailty terms were included to account for data clustering. For sensitivity analyses, we evaluated associations between MPA-AUC_0-12h_ and relapse rate, time-to-relapse, and time-to-B cell repopulation, restricting to rituximab-treated children.

To propose exposure MPA-AUC_0-12h_ targets, we modeled the association between MPA-AUC_0-12h_ and nephrotic syndrome relapse rate using GEE with Poisson links and natural splines. We created models with 1 to 4 knots and compared model fit using quasi-likelihood information criteria. We modelled anemia, leukopenia, neutropenia, thrombocytopenia, transaminitis, and hypercholesterolemia using a GEE with a binominal link to determine the predicted risk by MPA-AUC_0-12h_. Receiver operating characteristics–AUC plots with Youden indices (threshold where the sum of prediction sensitivity and specificity is greatest) were used to propose exposure MPA-AUC_0-12h_ thresholds for prediction of relapse and neutropenia occurrence during the observation window. Relapse and adverse effect outcomes were reported using descriptive statistics, stratified by MPA-AUC_0-12h_ (< 40, 40–59, or ≥ 60 mg h/l) and compared using distribution-appropriate hypothesis tests. These MPA-AUC_0-12h_ categories were chosen based on their associations with relapse rate and neutropenia.

## Results

### Participant Characteristics

Between 2019 and 2025, 131 MPA-AUC_0-12h_ measurements were performed among 67 children diagnosed with nephrotic syndrome on MMF or EC-MPS treatment (Table 1). Of these 67 children, 48 (72%) were male, median age at diagnosis was 3.6 years (interquartile range [IQR], 2.5–5.2). Four children (6%) were initially steroid resistant but subsequently became treatment responsive. Forty-one children (61%) were treated with EC-MPS and 26 (39%) with MMF. Fifty-eight (87%) received MMF concomitantly with rituximab. Before initiating MPA therapy, 61 children (91%) had previously received tacrolimus, and 25 (37%) had previously received cyclophosphamide. Median time from the last rituximab dose to the start of each MPA observation window was 1.5 (IQR, 0.9–3.9) months. The first MPA-AUC_0-12h_ measurement was performed at a median of 4.3 (IQR, 2.8–9.5) years from nephrotic syndrome diagnosis, and a median of 2.3 (IQR, 1.5–3.5) months after initiating MPA therapy.

**Table 1:** Characteristics of 67 children diagnosed with nephrotic syndrome that underwent MPA-AUC_0-12h_ monitoring

Participant characteristic	Overall cohort, *N* = 67
Male sex, *n* (%)	48 (72)
Age at diagnosis in yrs, median (IQR)	3.6 (2.5–5.2)
Yrs from diagnosis to first MPA-AUC_0-12h_, median (IQR)	4.3 (2.8–9.5)
Ethnicity, *n* (%)	
European	16 (24)
South Asian	17 (25)
East/Southeast Asian	9 (13)
Middle Eastern	23 (34)
Other	2 (3)
Nephrotic syndrome classification by 1-yr after diagnosis, *n* (%)	
Infrequent relapses	13 (19)
FRNS	10 (15)
FRNS + SDNS	40 (80)
SRNS^a^	4 (6)
Kidney biopsy histopathology, *n* (%)	
No biopsy performed	8 (12)
Minimal change disease	44 (66)
FSGS	9 (13)
Other	6 (9)
Prior cyclophosphamide, *n* (%)	25 (37)
Prior tacrolimus, *n* (%)	61 (91)
Prior rituximab, *n* (%)	58 (87)
Number of MPA-AUC_0-12h_ measurements per patient, *n* (%)	
1	36 (54)
2	14 (21)
3	9 (13)
≥ 4	8 (12)

AUC_0-12h_, area under the concentration-time curve over 12 h; FRNS, frequently relapsing nephrotic syndrome; FSGS, focal segmental glomerulosclerosis; IQR, interquartile range; MPA, mycophenolic acid; SDNS, steroid-dependent nephrotic syndrome; SRNS, steroid-resistant nephrotic syndrome.

a SRNS was defined as lack of complete remission after 4 to 6 wks of standard-dose steroids for the initial nephrotic syndrome episode. All SRNS cases were responsive to subsequent steroid-sparing immunosuppression.

### MPA Dose, Trough, and AUC_0-12h_

At the time of MPA-AUC_0-12h_ measurement, the median MMF equivalent dose was 1074 (IQR, 814–1163) mg/m^2^/d or 31.4 (IQR, 24.9–38.7) mg/kg/d. The median MPA-AUC_0-12h_ was 45.9 (IQR: 35.9–64.4) mg h/l, and the median MPA trough concentration was 2.76 (IQR, 1.61–4.41) mg/l. Weak correlations were observed between MPA-AUC_0-12h_ with MPA trough concentration (Figure 1a), total daily MMF equivalent dose in mg/m^2^/d (Figure 1b), and total daily MMF equivalent dose in mg/kg/d (Figure 1c).

**Figure 1:**
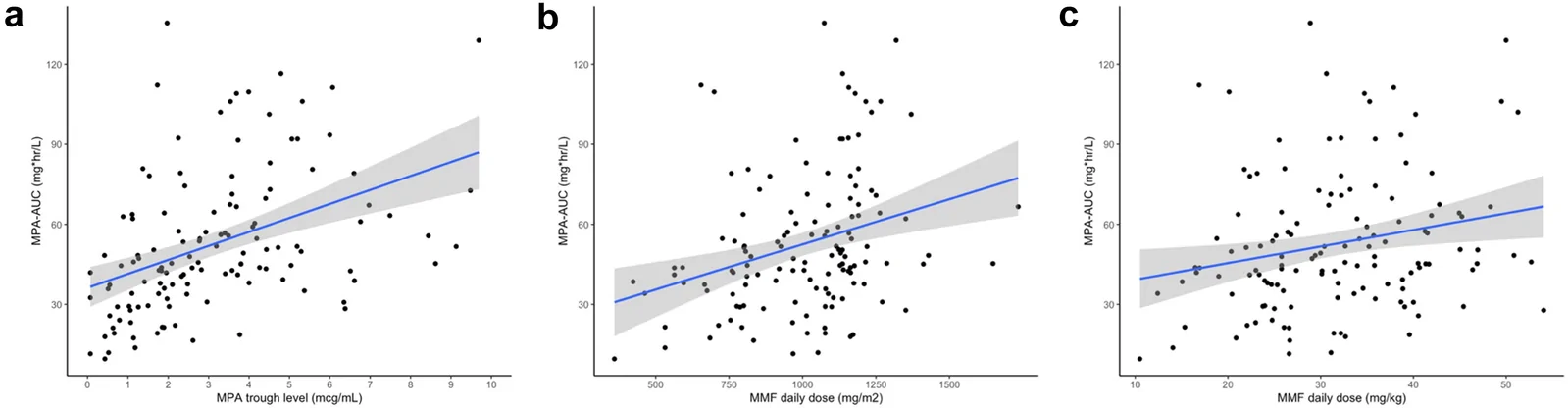
Scatterplots between MPA-AUC_0-12h_ and (a) MPA trough concentration, (b) total daily MMF equivalent dose in mg/m^2^, and (c) total daily MMF equivalent dose in mg/kg, among 131 mycophenolate observation windows. Weak correlations were observed between MPA-AUC_0-12h_ (mg h/l) and (a) MPA trough concentration in mg/l (Pearson correlation *r* = 0.39, *R*^2^ = 0.15), (b) total daily MMF equivalent dose in mg/m^2^/d (Pearson correlation *r* = 0.31, *R*^2^ = 0.09), and (c) total daily MMF equivalent dose in mg/kg/d (Pearson correlation *r* = 0.22, *R*^2^ = 0.05). AUC_0-12h_, area under the concentration-time curve over 12 hours; MMF, mycophenolate mofetil; MPA, mycophenolic acid.

### Associations Between MPA-AUC_0-12h_, MPA Trough Concentration, and MPA Dose With Relapses

Median follow-up for each MPA observation window (*N* = 131) was 6.9 (IQR: 2.9–16.3) months. Relapses occurred during 41 (31%) MPA observation windows. Mean relapse rate was 0.9 (SD, 1.9) relapses/person-year during each MPA observation window. The first relapse occurred at a median of 3.7 (IQR, 1.5–7.2) months after the start of the MPA observation window. Higher MPA-AUC_0-12h_ was associated with a significantly lower relapse rate (IRR, 0.70 per 10 mg h/l increase; 95% CI, 0.59–0.82; *P* < 0.001) and longer time-to-relapse (HR, 0.77 per 10 mg h/l increase; 95% CI, 0.64–0.92; *P* = 0.004; Table 2). No significant association was found between the time of AUC measurement and relapse rate (rate ratio, 0.99 [95% CI, 0.96–1.01; *P* = 0.18). A higher MPA-AUC_0-12h_ remains associated with a significantly lower relapse rate (IRR, 0.63 [95% CI, 0.44–0.90; *P* = 0.01]) when analysis was restricted to the first observation window. MPA-AUC_0-12h_ remains significantly associated with relapse rate when restricted to 25 children who received prior cyclophosphamide (IRR, 0.32; 95% CI, 0.15–0.67; *P* = 0.002) and 61 children who received prior tacrolimus (IRR, 0.65; 95% CI, 0.47–0.90; *P* = 0.009). MPA trough concentration was not significantly associated with relapse rate or time-to-relapse. Higher MMF equivalent dose in mg/m^2^/d and mg/kg/d were associated with borderline significant lower relapse rates but not time-to-relapse (Table 2). Stratifying MPA-AUC_0-12h_ by quartile (Table 3), we observed a strong, graded association between higher MPA-AUC_0-12h_ and lower relapse rate (*P* < 0.001 for trend). Increasing MPA trough concentration quartile was associated with a lower relapse rate (*P* = 0.02 for trend). In contrast, there was no significant trend observed between MMF equivalent dose in mg/m^2^ or mg/kg quartiles and relapse rates.

**Table 2:** Association between MPA-AUC_0-12h_, MPA trough concentration, and daily MMF equivalent dose with relapse outcomes

Outcome	Unadjusted estimate (95% CI)	*P*-value
MPA-AUC_0-12h_ (per 10 mg∗h/l increase)		
Relapse rate (relapses/yr)	IRR, 0.70 (0.59–0.82)	< 0.001
Time-to-relapse	HR, 0.77 (0.64–0.92)	0.004
MPA trough concentration (per 1 mg/l increase)		
Relapse rate (relapses/yr)	IRR, 0.86 (0.66–1.12)	0.28
Time-to-relapse	HR, 0.92 (0.71–1.19)	0.53
MMF equivalent dose (per 100 mg/m^2^ increase)		
Relapse rate (relapses/yr)	IRR, 0.86 (0.75–0.99)	0.04
Time-to-relapse	HR, 0.95 (0.81–1.11)	0.53
MMF equivalent dose (per 10 mg/kg increase)		
Relapse rate (relapses/yr)	IRR, 0.70 (0.49–1.00)	0.05
Time-to-relapse	HR, 0.82 (0.59–1.14)	0.25

95% CI, 95% confidence interval; AUC_0-12h_, area under the concentration-time curve over 12 h; HR, hazard ratio; IRR, incidence rate ratio; MMF, mycophenolate mofetil; MPA, mycophenolic acid.

**Table 3:** Association between MPA-AUC_0-12h_, MPA trough concentration, and daily MMF equivalent dose quartiles with relapse rate

Quartile	Relapse rate, RR (95% CI)	*P*-value	*P*-value for trend
MPA-AUC (mg-h/l)			
Quartile 1 (0–36)	Ref	Ref	
Quartile 2 (36–46)	0.46 (0.21–1.03)	0.06	< 0.001
Quartile 3 (46–64)	0.32 (0.13–0.75)	0.01	
Quartile 4 (≥ 64)	0.20 (0.08–0.52)	< 0.001	
MPA trough concentration (mg/l)			
Quartile 1 (0–1.6)	Ref	Ref	
Quartile 2 (1.6–2.8)	0.82 (0.36–1.87)	0.63	0.02
Quartile 3 (2.8–4.4)	0.28 (0.10–0.74)	0.01	
Quartile 4 (≥ 4.4)	0.40 (0.14–1.11)	0.08	
MMF equivalent dose (mg/m^2^/d)			
Quartile 1 (0–814)	Ref	Ref	
Quartile 2 (814–1074)	0.49 (0.20–1.21)	0.12	0.06
Quartile 3 (1074–1163)	0.55 (0.21–1.43)	0.22	
Quartile 4 (≥ 1163)	0.38 (0.16–0.89)	0.03	
MMF equivalent dose (mg/kg/d)			
Quartile 1 (0–25)	Ref	Ref	
Quartile 2 (25–31)	1.02 (0.40–2.61)	0.96	0.07
Quartile 3 (31–39)	0.83 (0.33–2.09)	0.70	
Quartile 4 (≥ 39)	0.40 (0.15–1.07)	0.07	

95% CI, 95% confidence interval; AUC_0-12h_, area under the concentration-time curve over 12 h, MMF, mycophenolate mofetil; MPA, mycophenolic acid; Ref, reference; RR, rate ratio.

In sensitivity analyses restricting to 119 MPA observation windows among 58 (87%) children previously treated with rituximab, higher MPA-AUC_0-12h_ remained associated with a significantly lower relapse rate (IRR, 0.69 per 10 mg h/l increase; 95% CI, 0.58–0.83) and longer time-to-relapse (HR, 0.76 per 10 mg h/l increase; 95% CI, 0.61–0.94; Supplementary Table S1). Similar trends were observed for stratifying MPA-AUC_0-12h_ by quartile (Supplementary Table S2). Median time-to-B-cell repopulation after rituximab was 5.1 (IQR, 4.1–6.9) months. There was no difference in time-to-B cell repopulation based on MPA-AUC_0-12h_ (HR, 0.98 per 10 mg h/l increase; 95% CI, 0.91–1.05; *P* = 0.58; Supplementary Figure S1). Relapses occurred during 34 (29%) MPA observation windows. Mean relapse rate was 0.9 (SD, 1.9) relapses/person-year during each MPA observation window. Relapses occurred in 25 children (74%) after B-cell repopulation, at a median of 5.5 (IQR, 2–10) months since B-cell repopulation. Although MPA-AUC_0-12h_ was lower among children who relapsed while B-cells were depleted (median, 36.6 mg h/l; IQR, 26.9–45.8) than children who relapsed after B-cell repopulation (median, 44.9 mg h/l; IQR, 35.1–54.8), this difference was not statistically significant (*P* = 0.12).

### MPA-AUC_0-12h_ Target for Efficacy

Using Poisson regression models with natural splines to evaluate the association between MPA-AUC_0-12h_ and relapse rate, a model with 2 knots at MPA-AUC_0-12h_ of 41 mg h/l and 57 mg h/l achieved the best model fit (Figure 2). Similar results (MPA-AUC_0-12h_ knots at 41 and 59 mg h/l) were found in a sensitivity analysis restricted to 119 MPA observation windows among children previously treated with rituximab (Supplementary Figure S2). The receiver operating characteristics–AUC of MPA-AUC_0-12h_ for predicting relapse occurrence (≥ 1 relapse during the observation window) was 0.65, with a Youden index of 71 mg h/l (Supplementary Figure S3A). Based on these results, we categorized MPA-AUC_0-12h_ as < 40 mg,h/l (*n* = 42), 40 to 59 mg h/l (*n* = 49), and ≥ 60 mg h/l (*n* = 40; Table 4). Relapse rate was significantly lower among children with an MPA-AUC_0-12h_ ≥ 60 mg h/l (IRR, 0.23; 95% CI, 0.10–0.55) or MPA-AUC_0-12h_ of 40 to 59 mg h/l (IRR, 0.32; 95% CI, 0.17–0.62), when compared with children with an MPA-AUC_0-12h_ < 40 mg h/l. Relapses occurred in 16 (38%) children with an MPA-AUC_0-12h_ < 40 mg h/l, 19 (39%) children with an MPA-AUC_0-12h_ of 40 to 59 mg h/l, and 6 (15%) children with an MPA-AUC_0-12h_ ≥ 60 mg h/l. Relapse-free survival was significantly longer among children with an MPA-AUC_0-12h_ > 40 mg h/l, compared with those with MPA-AUC_0-12h_ < 40 mg h/l (HR, 0.51; 95% CI, 0.28–0.93; *P* = 0.03; Figure 3).

**Figure 2:**
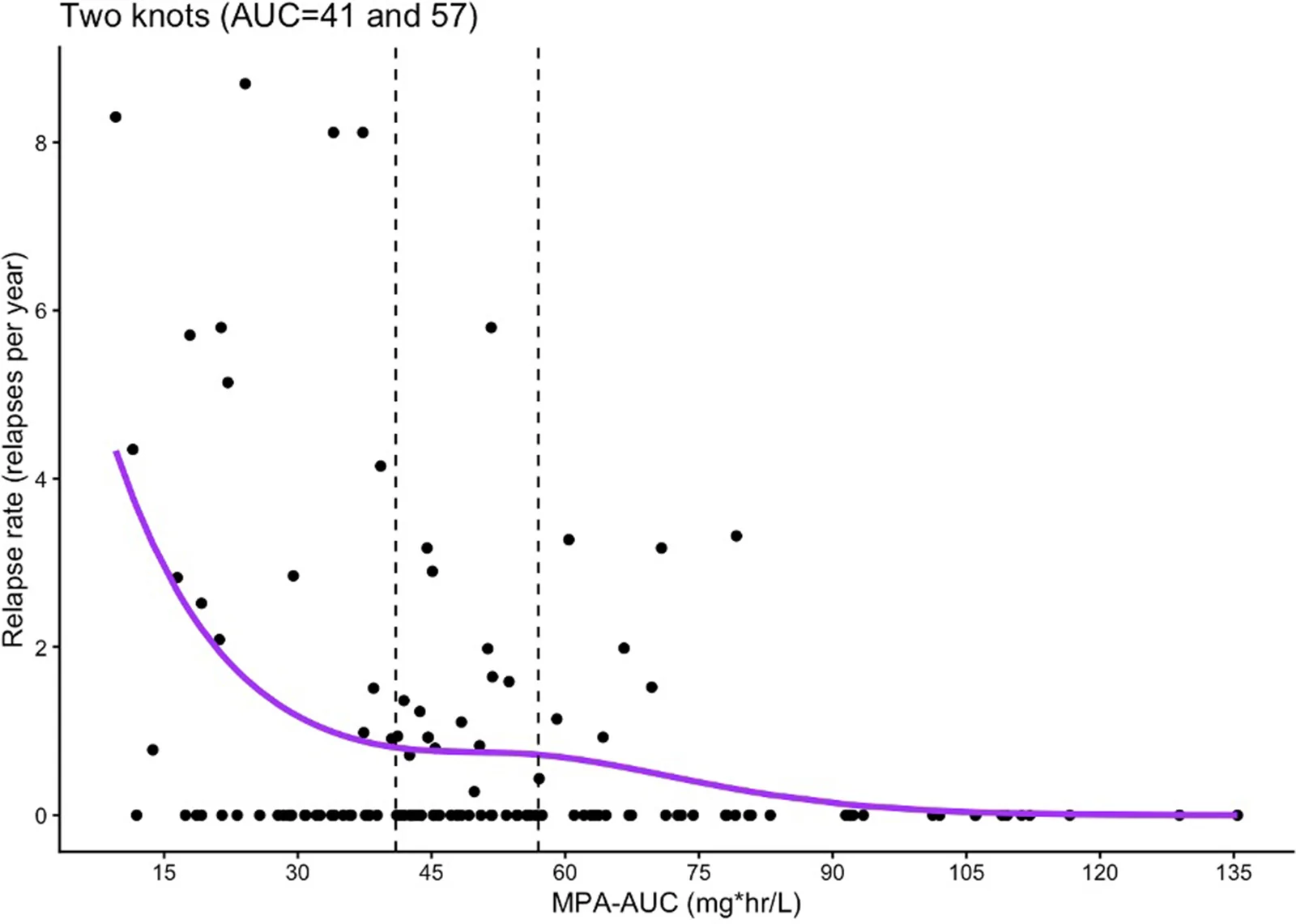
Association between MPA-AUC_0-12h_ and nephrotic syndrome relapse rate, among 131 mycophenolate observation windows. Splines model with 2 knots achieved the best model fit (quasi-likelihood information criterion = 198), compared with models with 1 knot (quasi-likelihood information criterion = 202), 3 knots (quasi-likelihood information criterion = 199), or 4 knots (quasi-likelihood information criterion = 200). AUC_0-12h_, area under the concentration-time curve over 12 hours; MPA, mycophenolic acid.

**Table 4:** Outcomes among 131 MPA observation windows, stratified by MPA-AUC_0-12h_ thresholds

Characteristic or outcome	MPA-AUC_0-12h_ < 40 mg h/l, *n* = 42	MPA-AUC_0-12h_ 40–59 mg h/l, *n* = 49	MPA-AUC_0-12h_ ≥ 60 mg h/l, *n* = 40	*P*-value
Participant characteristics				
MPA-AUC in mg h/l, median (IQR)	29.1 (21.2–34.9)	47.2 (43.7–51.8)	79.1 (67.4–101.4)	< 0.001
MMF dose equivalent in mg/m^2^/d, median (IQR)	967 (810–1495)	1075 (824–1145)	1157 (1004–1197)	< 0.001
Age at MPA-AUC, median in yrs (IQR)	9.0 (7.1–13.8)	12.0 (7.0–14.0)	10.5 (7.8–15.0)	0.7
Male sex, *n* (%)	27 (64)	36 (74)	29 (73)	0.6
Nephrotic syndrome classification by 1-yr after diagnosis, *n* (%)				
Infrequent relapses	8 (19)	12 (25)	9 (23)	0.6
FRNS/SDNS	30 (71)	26 (74)	29 (73)	
SRNS	4 (10)	1 (2)	2 (5)	
Relapse outcomes				
Follow-up duration in months, median (IQR)	4.2 (2.3–9.0)	15.0 (7.5–26.3)	3.6 (1.8–8.4)	< 0.001
≥1 relapse, *n* (%)	16 (38)	19 (39)	6 (15)	0.03
Relapse rate/person-year, mean (SD)	1.7 (2.8)	0.6 (1.1)	0.4 (0.9)	0.001
Time to relapse in mos, median (IQR)	2.5 (1.4–5.1)	6.8 (4.4–13.6)	1.8 (1.2–2.2)	0.02
Safety outcomes				
Anemia, *n* (%)	1 (2)	3 (6)	2 (3)	0.7
Leukopenia, *n* (%)	1 (2)	8 (16)	1 (3)	0.02
Neutropenia, *n* (%)	2 (5)	8 (16)	9 (23)	0.07
Thrombocytopenia, *n* (%)	0	1 (2)	0	0.4
Transaminitis, *n* (%)	2 (5)	6 (12)	0	0.05
Hypercholesterolemia, *n* (%)	13 (31)	18 (37)	11 (28)	0.6

AUC_0-12h_, area under the concentration-time curve over 12 h; FRNS, frequently relapsing nephrotic syndrome; IQR, interquartile range; MMF, mycophenolate mofetil; MPA, mycophenolic acid; SDNS, steroid-dependent nephrotic syndrome; SMD, standardized mean difference, SRNS, steroid-resistant nephrotic syndrome.

**Figure 3:**
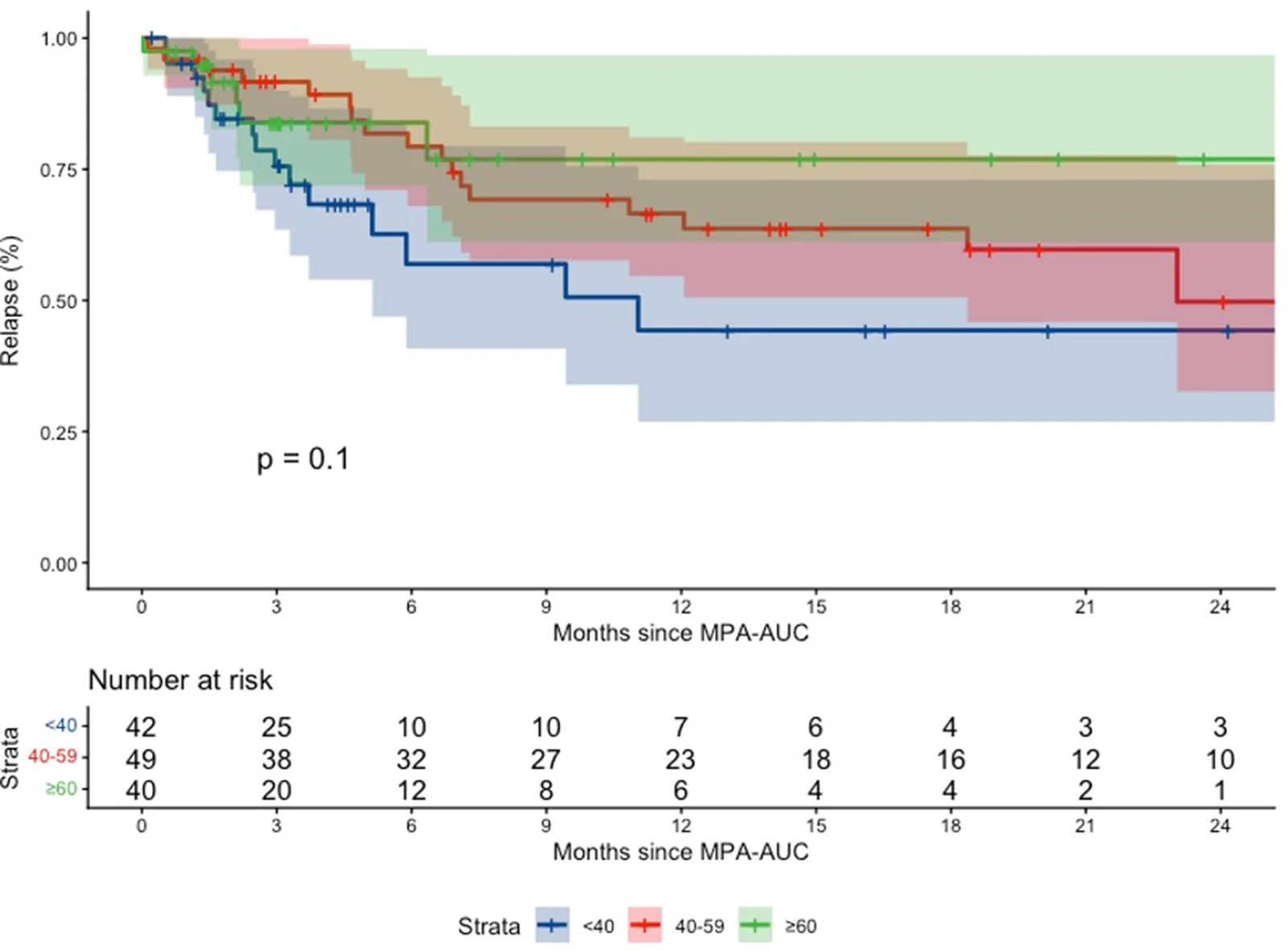
Association between MPA-AUC_0-12h_ and time to nephrotic syndrome relapse, among 131 mycophenolate observation windows. Kaplan-Meier curve for relapse-free survival stratified by MPA-AUC_0-12h_ (mg h/l) of < 40 (blue), 40 to 59 (red), and ≥ 60 (green). The shaded area represents the confidence interval. AUC_0-12h_, area under the concentration-time curve over 12 hours; MPA, mycophenolic acid.

### MPA-AUC_0-12h_ Target to Minimize Adverse Effects

During follow-up, anemia occurred in 6 children (5%), leukopenia in 10 children (8%), neutropenia in 19 children (15%), thrombocytopenia in 1 child (1%), transaminitis in 8 children (6%), and hypercholesterolemia in 42 children (32%). There were no substantial differences in the frequency of anemia, thrombocytopenia, transaminitis, or hypercholesterolemia by MPA-AUC_0-12h_ (Table 4). Neutropenia was found to be more common among children with an MPA-AUC_0-12h_ ≥ 60 mg h/l (23%) and 40 to 59 mg h/l (16%), than among children with an MPA-AUC_0-12h_ < 40 mg h/l (5%; Table 4, Supplementary Table S3 for patients who previously received rituximab). The risk of neutropenia increased significantly with higher MPA-AUC_0-12h_ (odds ratio, 1.18 per 10 mg h/l increase; 95% CI, 1.02–1.36; *P* = 0.03). Time to neutropenia was significantly shorter among children with an MPA-AUC_0-12h_ ≥ 60 mg h/l, compared with those with MPA-AUC_0-12h_ < 60 mg h/l (Figure 4). The receiver operating characteristics–AUC of MPA-AUC_0-12h_ for predicting neutropenia occurrence was 0.66, with a Youden index of 42 mg h/l (Supplementary Figure S3B). MPA-AUC_0-12h_ and MMF equivalent dose (mg/kg) showed the strongest associations with neutropenia (Supplementary Table S4). Comparing children who did not develop neutropenia (*n* = 112) to those who did (*n* = 19), there were no substantial differences in time since last rituximab dose (median, 1.5 months vs. 2 months; *P* = 0.4) or number of prior rituximab doses (median: 3 doses each). Of the 19 children who developed neutropenia, 13 were mild (absolute neutrophil count: 1.0–1.5 × 10^9^/l), 4 were moderate (absolute neutrophil count: 0.5–1.0 × 10^9^/l), and 2 were severe (absolute neutrophil count: < 0.5 × 10^9^/l). Among those with moderate or severe neutropenia, MPA-AUC_0-12h_ was ≥ 60 in 2, 40 to 59 in 3, and < 40 mg h/l in 1 child. Neutropenia resolved spontaneously while continuing MPA in 2, with MPA dose reduction in 1, and after holding MPA in 3 of these children. No serious infections occurred, and MPA was restarted without recurrence of neutropenia in all 3 children in whom it had been held.

**Figure 4:**
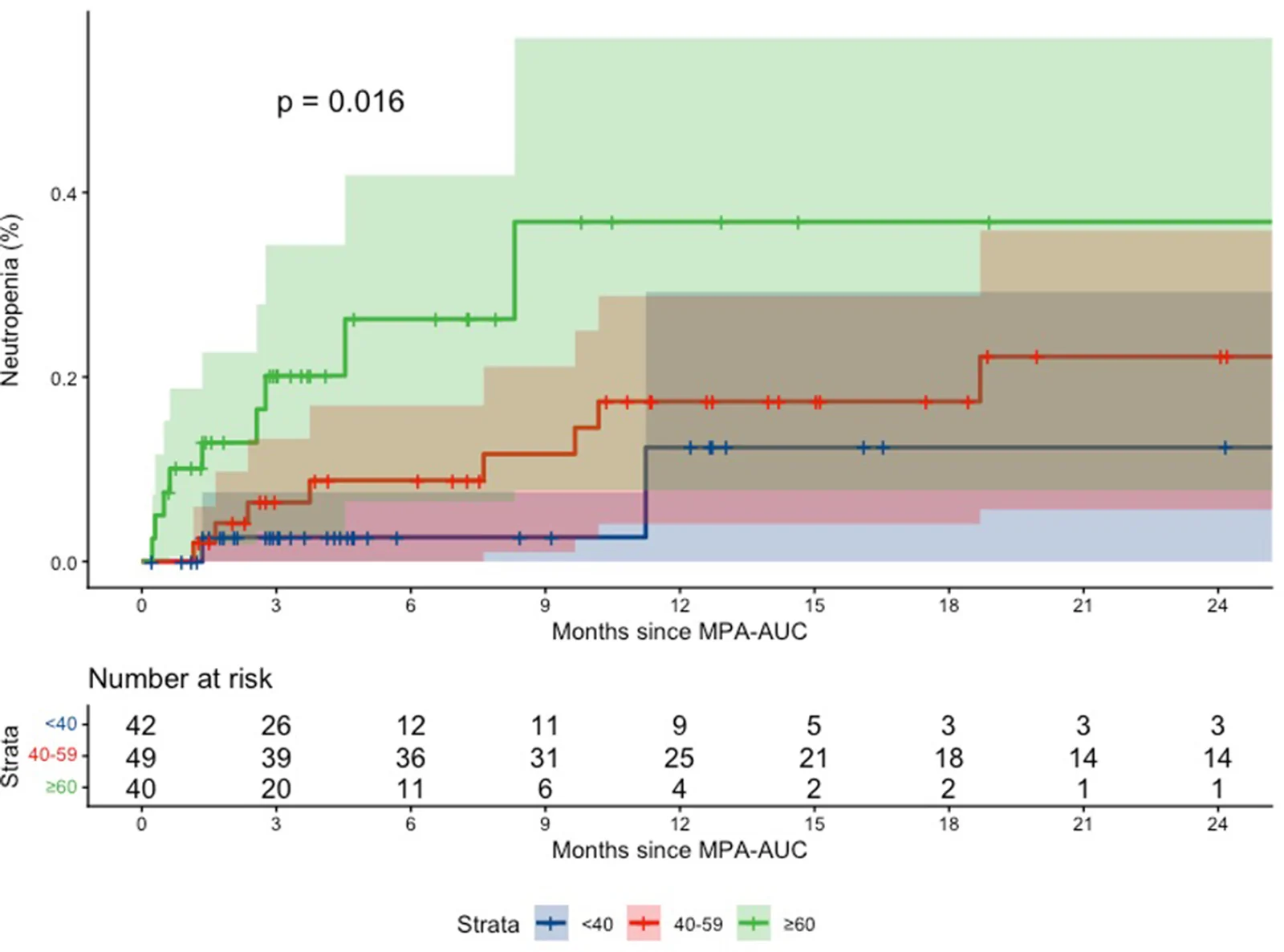
Association between MPA-AUC_0-12h_ and time to neutropenia, among 131 mycophenolate observation windows. Kaplan-Meier curve for time to neutropenia stratified by MPA-AUC_0-12h_ (mg h/l) of < 40 (blue), 40 to 59 (red), and ≥ 60 (green). The shaded area represents the confidence interval. AUC_0-12h_, area under the concentration-time curve over 12 hours; MPA, mycophenolic acid.

## Discussion

Children with nephrotic syndrome experience a high disease burden with multiple relapses and steroid-related toxicity. MPA, especially in conjunction with rituximab, is a promising therapy to prolong remission for patients with FRNS.^13^, ^36^ TDM of MPA when used concomitantly with rituximab is not well-established in nephrotic syndrome. Among 67 children with immunosuppressive-responsive nephrotic syndrome who predominantly initiated MPA in conjunction with rituximab therapy, we found that higher MPA-AUC_0-12h_ was associated with lower relapse rate and prolonged relapse-free survival, but higher risk of neutropenia. Each 10 mg h/l increase in MPA-AUC_0-12h_ was associated with 30% lower relapse rate, 23% longer relapse-free survival, but 18% higher odds of neutropenia. Our findings suggest that an initial MPA-AUC_0-12h_ target of 40 to 60 mg h/l may provide a favorable balance between relapse prevention and adverse effects among children with nephrotic syndrome. A higher MPA-AUC_0-12h_ target (e.g., ≥ 60 mg h/l) could provide additional benefit in terms of relapse prevention; however, this must be weighed against the increased risk of adverse effects, including neutropenia.

Due to the lack of well-established MPA-AUC_0-12h_ targets in nephrotic syndrome, physicians often dose by body weight or surface area and adjust based on symptoms or laboratory abnormalities.^19^ However, we and others have shown that dosing by body weight or surface area is only weakly correlated with MPA-AUC_0-12h_.^10^, ^37^, ^38^ In our study, we did not find a correlation between dose by body weight or surface area and relapse rate or time-to-relapse. MPA trough concentrations have previously been shown to correlate with a lower relapse rate.^19^ Although MPA trough concentrations are a more convenient and practical means for patient monitoring, we found that trough concentrations were only weakly correlated with MPA-AUC_0-12h_ and were not significantly associated with relapse rate or time-to-relapse.^19^ In addition, assessing MPA-AUC_0-12h_ is superior to measuring MPA trough concentrations because it provides a complete pharmacokinetic profile of MPA, including enterohepatic recirculation. MMF undergoes rapid and extensive absorption to become MPA, with varying bioavailability between MMF and EC-MPS, and achieves maximum concentration within 1 hour. Furthermore, MPA undergoes enterohepatic recirculation, with its plasma concentration showing a secondary peak concentration 6 to 12 hours after dose administration, which collectively contributes to the overall AUC.^15^, ^33^, ^34^ Thus, whenever possible, measuring MPA-AUC_0-12h_ should be considered for determining TDM in patients with nephrotic syndrome on MPA.^7^, ^20^, ^38^

Most children in prior studies received MPA without concomitant rituximab. Our study is the first to establish MPA-AUC_0-12h_ targets in patients receiving MPA as concomitant immunosuppression with rituximab. Sensitivity analyses restricted to patients receiving MPA concomitantly with rituximab (87% of the total cohort) showed similar trends. Unfortunately, our cohorts were too small to compare patients receiving MPA alone with those receiving MPA concomitantly with rituximab. In addition, this is the first study to assess associations between MPA-AUC_0-12h_ and adverse effects, an important factor in determining the upper MPA-AUC_0-12h_ target threshold, especially in patients receiving concomitant rituximab. Our analysis found an 18% increased odds for neutropenia for every 10 mg h/l increase in MPA-AUC_0-12h_. We found no significant associations between MPA-AUC_0-12h_ and other hematological abnormalities or transaminitis.

The International Pediatric Nephrology Association guidelines for patients with steroid-sensitive nephrotic syndrome suggest targeting MPA-AUC_0-12h_ > 50 mg h/l.^7^ This was based on a *post hoc* analysis of data from a 2013 trial comparing MMF (dosed at 1000–1200 mg/m^2^/d) with cyclosporine A. Although cyclosporine A was associated with longer relapse-free survival than MMF overall, there was no difference when compared with children with an MPA AUC_0-12h_ > 50 mg h/l.^10^ Other studies similarly have defined a lower MPA-AUC_0-12h_ threshold in nephrotic syndrome of 45 or 50 mg h/l to be associated with a lower relapse risk.^10^, ^16^, ^17^, ^39^ Our study corroborates these findings, but additionally suggests that further increases in MPA-AUC_0-12h_ (i.e., targeting MPA-AUC_0-12h_ ≥ 60 mg h/l) may provide further reduction of relapse risk. However, this should be carefully weighed against the risk of neutropenia. These findings would suggest a degree of caution in the interpretation that MPA is inferior at relapse prevention when compared with other steroid-sparing immunosuppressants in previous clinical trials that aimed for lower MPA-AUC_0-12h_ or did not include TDM.^8^, ^10^, ^21^, ^40^, ^41^, ^42^ Based on these findings, future trials studying the efficacy of MPA in nephrotic syndrome should include TDM, aiming for MPA-AUC_0-12h_ ≥ 40 to 60 mg h/l.

A key consideration in applying TDM clinically is the sampling strategy used to estimate MPA-AUC. Limited sampling strategies for MMF should be preferred over an intensive pharmacokinetic sampling approach using ≥ 7 samples, because they reduce the number of blood samples, and overall time and cost.^10^, ^16^, ^17^, ^18^, ^39^ The International Pediatric Nephrology Association guideline suggests using LSS used in Benz *et al.*^43^ (validated in small nephrotic syndrome cohort) or Pawinski *et al.*^44^ (validated in adult heart transplant patients with concomitant cyclosporine A). Differences in the pharmacokinetic profile between posttransplant and nephrotic syndrome patients have previously been described.^45^ Although Benz *et al.*^43^ has been validated in patients with nephrotic syndrome, this formula only includes short time points and no later time points (e.g., 6–12 hours) to assess the potential effect of enterohepatic recirculation. Enterohepatic recirculation can affect the overall MPA-AUC_0-12h_, and has been shown to vary substantially between individuals and between measurements in the same individual.^33^, ^34^, ^46^ In our study, we used an LSS equation validated in the transplant population that included a later time point (6h) in order to account for enterohepatic recirculation. Further studies are needed to determine the “optimal” LSS equation in patients with nephrotic syndrome treated with MMF.

The majority of children in our study received EC-MPS. When prescribed at bioequivalent doses (MMF 1000mg equals EC-MPS 720 mg^35^) in transplant patients, the same overall drug exposure, estimated through MPA-AUC was observed. However, the delayed-release nature of EC-MPS leads to different absorption dynamics and higher variability, making limited sampling strategies more challenging. Further validation of limited sampling strategies, or model-informed precision dosing using population pharmacokinetics for patients with nephrotic syndrome on EC-MPS would be helpful to minimize the burden for patients and families.^47^

The limitations of this study include the retrospective nature and single-center design, which may limit generalizability to other populations. MPA-AUC_0-12h_ measurement at a single time point for each observation window may not accurately reflect drug exposure throughout that time. MPA-AUC_0-12h_ was measured after initiation and dose changes. MMF dose adjustments were made by the clinical team, based on MPA-AUC_0-12h_ levels and on clinical outcomes (e.g., suboptimal nephrotic syndrome remission maintenance and/or adverse effects). This approach, albeit clinically relevant and reflective of real-world practice, might have resulted in more AUC_0-12h_ measurements and dose adjustments in patients with the lowest and highest MPA-AUC_0-12h_ measurements. We used limited sampling strategies followed by a linear regression equation to derive the MPA-AUC_0-12h_, which was extrapolated from the transplant literature.^28^ We were unable to determine the rate of gastrointestinal side effects and other patient-reported side effects, for example, minor or mild infections, due to inconsistent documentation. Future studies might be helpful to correlate free MPA-AUC with total MPA-AUC_0-12h_, especially in patients with nephrotic syndrome where the protein-binding capacity of MPA might vary and previous case reports have indicated that elevated free MPA-AUC is associated with side effects.^48^, ^49^

MPA is an important steroid-sparing agent in childhood nephrotic syndrome. MPA-AUC_0-12h_ is recommended to ensure appropriate drug exposure, and higher MPA-AUC_0-12h_ is associated with a lower rate of nephrotic syndrome relapses but increased risk of neutropenia. Targeting an initial MPA-AUC_0-12h_ between 40 and 60 mg h/l may provide a more favorable balance between relapse prevention and adverse effects. Further increases of MPA-AUC_0-12h_ to > 60 mg h/l may provide further relapse risk reduction; however, that should be carefully weighed against the risk of neutropenia.

## Disclosures

All authors declared no competing interests.

## Supplementary Material

Supplementary File (PDF) Figure S1. Association between MPA-AUC_0-12h_ and time to B-cell repopulation, among 119 mycophenolate observation windows in children that previously received rituximab. Figure S2. Association between MPA-AUC_0-12h_ and nephrotic syndrome relapse rate, among 119 mycophenolate observation windows in children that received prior rituximab treatment. Figure S3. Youden index for optimal MPA-AUC_0-12h_ to predict (A) a relapse occurrence and (B) neutropenia occurrence. Table S1. Association between MPA-AUC_0-12h_, MPA trough concentration, and daily MMF equivalent dose with relapse among 119 mycophenolate observation windows in children that previously received rituximab. Table S2. Association between MPA-AUC_0-12h_, MPA trough concentration, and daily MMF equivalent dose quartiles with relapse rate among 119 mycophenolate observation windows in children that previously received rituximab. Table S3. Outcomes among 119 mycophenolate observation windows in children that previously received rituximab, stratified by MPA-AUC_0-12h_ thresholds. Table S4, Association between MPA-AUC_0-12h_, MPA trough concentration, and daily MMF equivalent dose with neutropenia. STROBE checklist.

## Footnotes

Supplementary File (PDF)
**Figure S1.** Association between MPA-AUC_0-12h_ and time to B-cell repopulation, among 119 mycophenolate observation windows in children that previously received rituximab. **Figure S2.** Association between MPA-AUC_0-12h_ and nephrotic syndrome relapse rate, among 119 mycophenolate observation windows in children that received prior rituximab treatment. **Figure S3.** Youden index for optimal MPA-AUC_0-12h_ to predict (A) relapse occurrence and (B) neutropenia occurrence. **Table S1.** Association between MPA-AUC_0-12h_, MPA trough concentration, and daily MMF equivalent dose with relapse outcomes among 119 mycophenolate observation windows in children that previously received rituximab. **Table S2.** Association between MPA-AUC_0-12h_, MPA trough concentration, and daily MMF equivalent dose quartiles with relapse rate among 119 mycophenolate observation windows in children that previously received rituximab. **Table S3.** Outcomes among 119 mycophenolate observation windows in children that previously received rituximab, stratified by MPA-AUC_0-12h_ thresholds. **Table S4,** Association between MPA-AUC_0-12h_, MPA trough concentration, and daily MMF equivalent dose with neutropenia. STROBE checklist.
