# Supplementary material for: Establishing Mycophenolate Therapeutic Drug Monitoring Targets in Childhood Nephrotic Syndrome
Source: Kidney Int Rep. 2026 Oct 6;11(12):107131. doi: 10.1016/j.ekir.2026.107131 (PMC13755566; doi:10.1016/j.ekir.2026.107131)
Supplement: Supplementary file 1 [file mmc1.pdf]

Supplementary Material

Supplementary File (PDF)

- Supplementary Table S1
- Supplementary Table S2
- Supplementary Table S3
- Supplementary Table S4
- Supplementary Figure S1
- Supplementary Figure S2
- Supplementary Figure S3
- STROBE checklist

Supplementary information is available at KI Report's website.

Supplemental Table S1. Association between MPA-$AUC_{0\text{-}12h}$, MPA trough concentration, and daily MMF equivalent dose with relapse outcomes among 119 mycophenolate observation windows in children that previously received rituximab

Outcome	Unadjusted estimate (95% CI)	p-value
**MPA-$AUC_{0\text{-}12h}$ (per 10 mg*hr/L increase)**		
Relapse rate (relapses per year)	IRR 0.69 (0.58-0.83)	<0.001
Time-to-relapse	HR 0.76 (0.61-0.94)	0.01
**MPA trough concentration (per 1 mg/L increase)**		
Relapse rate (relapses per year)	IRR 0.88 (0.65-1.19)	0.39
Time-to-relapse	HR 0.97 (0.74-1.26)	0.80
**MMF equivalent dose (per 100 mg/m^2 increase)**		
Relapse rate (relapses per year)	IRR 0.83 (0.72-0.96)	0.01
Time-to-relapse	HR 0.91 (0.77-1.08)	0.28
**MMF equivalent dose (per 10 mg/kg increase)**		
Relapse rate (relapses per year)	IRR 0.63 (0.42-0.93)	0.02
Time-to-relapse	HR 0.78 (0.53-1.15)	0.21

MPA: mycophenolic acid, $AUC_{0\text{-}12h}$: area under the concentration-time curve over 12 hours, MMF: mycophenolate mofetil, 95% CI: 95% confidence interval, IRR: incidence rate ratio, HR: hazard ratio

Supplemental Table S2. Association between MPA-AUC$_{0-12h}$, MPA trough concentration, and daily MMF equivalent dose quartiles with relapse rate among 119 mycophenolate observation windows in children that previously received rituximab

Quartile	Relapse rate, RR (95% CI)	p-value	p-value for trend
**MPA-AUC (mg.hr/L)**			
Quartile 1 (0-36)	Ref	Ref	0.004
Quartile 2 (36-46)	0.47 (0.20-1.08)	0.08	
Quartile 3 (46-67)	0.36 (0.15-0.86)	0.02	
Quartile 4 (≥67)	0.14 (0.04-0.53)	0.003	
**MPA trough concentration (mg/L)**			
Quartile 1 (0-1.6)	Ref	Ref	0.05
Quartile 2 (1.6-2.8)	0.83 (0.33-2.07)	0.69	
Quartile 3 (2.8-4.4)	0.30 (0.09-1.00)	0.05	
Quartile 4 (≥4.4)	0.44 (0.16-1.25)	0.12	
**MMF equivalent dose (mg/m^2/day)**			
Quartile 1 (0-814)	Ref	Ref	0.11
Quartile 2 (814-1074)	0.42 (0.16-1.12)	0.08	
Quartile 3 (1074-1163)	0.37 (0.11-1.25)	0.11	
Quartile 4 (≥1163)	0.43 (0.18-1.03)	0.06	
**MMF equivalent dose (mg/kg/day)**			
Quartile 1 (0-25)	Ref	Ref	0.04
Quartile 2 (25-32)	0.82 (0.30-2.20)	0.69	
Quartile 3 (32-39)	0.72 (0.24-2.15)	0.55	
Quartile 4 (≥39)	0.31 (0.10-0.91)	0.03	

MPA: mycophenolic acid, AUC$_{0-12h}$: area under the concentration-time curve over 12 hours, MMF: mycophenolate mofetil, 95% CI: 95% confidence interval, RR: rate ratio

Supplemental Table S3. Outcomes among 119 MPA observation windows in children that previously received rituximab, stratified by MPA-AUC$_{0-12h}$ thresholds

Characteristic or outcome	MPA-AUC$_{0-12h}$ <40 mg*hr/L, N=39	MPA-AUC$_{0-12h}$ 40-59 mg*hr/L, N=41	MPA-AUC$_{0-12h}$ ≥60 mg*hr/L, N=39	p-value
**Participant characteristics**				
MPA-AUC in mg*hr/L, median (IQR)	29.3 (21.8-35.5)	45.7 (43.7-51.8)	79.2 (68.6-101.6)	<0.001
MMF dose equivalent in mg/m^2/day, median (IQR)	966 (759-1078)	1075 (824-1145)	1156 (995-1191)	<0.001
Age at MPA-AUC, median in years (IQR)	9 (7-14)	11 (7-13)	10 (8-15)	0.6
Male sex, n (%)	24 (62%)	30 (73%)	28 (72%)	0.5
Nephrotic syndrome classification by 1-year after diagnosis, n (%)				0.7
Infrequent relapses	8 (21%)	8 (20%)	9 (23%)	
FRNS/SDNS	27 (69%)	32 (78%)	28 (72%)	
SRNS	4 (10%)	1 (2%)	2 (5%)	
**Relapse outcomes**				
Follow-up duration in months, median (IQR)	3.6 (2.1-8.6)	15.0 (7.5-26.3)	3.6 (1.7-7.9)	<0.001
≥1 relapse, n (%)	13 (33%)	16 (39%)	5 (13%)	0.03
Relapse rate per person-year, mean (SD)	1.6 (2.8)	0.6 (1.2)	0.3 (0.9)	0.005
Time to relapse in months, median (IQR)	2.1 (1.2-4.6)	6.3 (4.4-8.2)	1.5 (1.1-2.2)	0.03
**Safety outcomes** [1]				
Anemia, n (%)	1 (3%)	3 (7%)	2 (5%)	0.6
Leukopenia, n (%)	1 (3%)	7 (17%)	1 (3%)	0.02
Neutropenia, n (%)	2 (5%)	8 (20%)	8 (21%)	0.1
Thrombocytopenia, n (%)	0	1 (2%)	0	0.4
Transaminitis, n (%)	1 (3%)	6 (15%)	0	0.01
Hypercholesterolemia, n (%)	10 (26%)	13 (32%)	10 (26%)	0.8

MPA: mycophenolic acid, AUC$_{0-12h}$: area under the concentration-time curve over 12 hours, MMF: mycophenolate mofetil, SMD: standardized mean difference, IQR: interquartile range, FRNS: frequently relapsing nephrotic syndrome, SDNS: steroid dependent nephrotic syndrome, SRNS: steroid resistant nephrotic syndrome, SD: standard deviation

Supplemental Table S4. Association between MPA-$AUC_{0\text{-}12h}$, MPA trough concentration, and daily MMF equivalent dose with neutropenia

**Outcome**	**Unadjusted estimate (95% CI)**	**p-value**
**MPA-$AUC_{0\text{-}12h}$ (per 10 mg*hr/L increase)**		
Neutropenia occurrence	OR 1.18 (1.02-1.36)	0.03
**MPA trough concentration (per 1 mg/L increase)**		
Neutropenia occurrence	OR 0.98 (0.85-1.12)	0.74
**MMF equivalent dose (per 100 mg/m^2 increase)**		
Neutropenia occurrence	OR 1.07 (0.84-1.37)	0.56
**MMF equivalent dose (per 10 mg/kg increase)**		
Neutropenia occurrence	OR 1.67 (0.93-3.00)	0.09
MPA: mycophenolic acid, $AUC_{0\text{-}12h}$: area under the concentration-time curve over 12 hours, MMF: mycophenolate mofetil, 95% CI: 95% confidence interval, OR: odds ratio		

Supplemental Figure S1. Association between MPA-AUC$_{0\text{-}12h}$ and time to B cell repopulation, among 119 mycophenolate observation windows in children that previously received rituximab

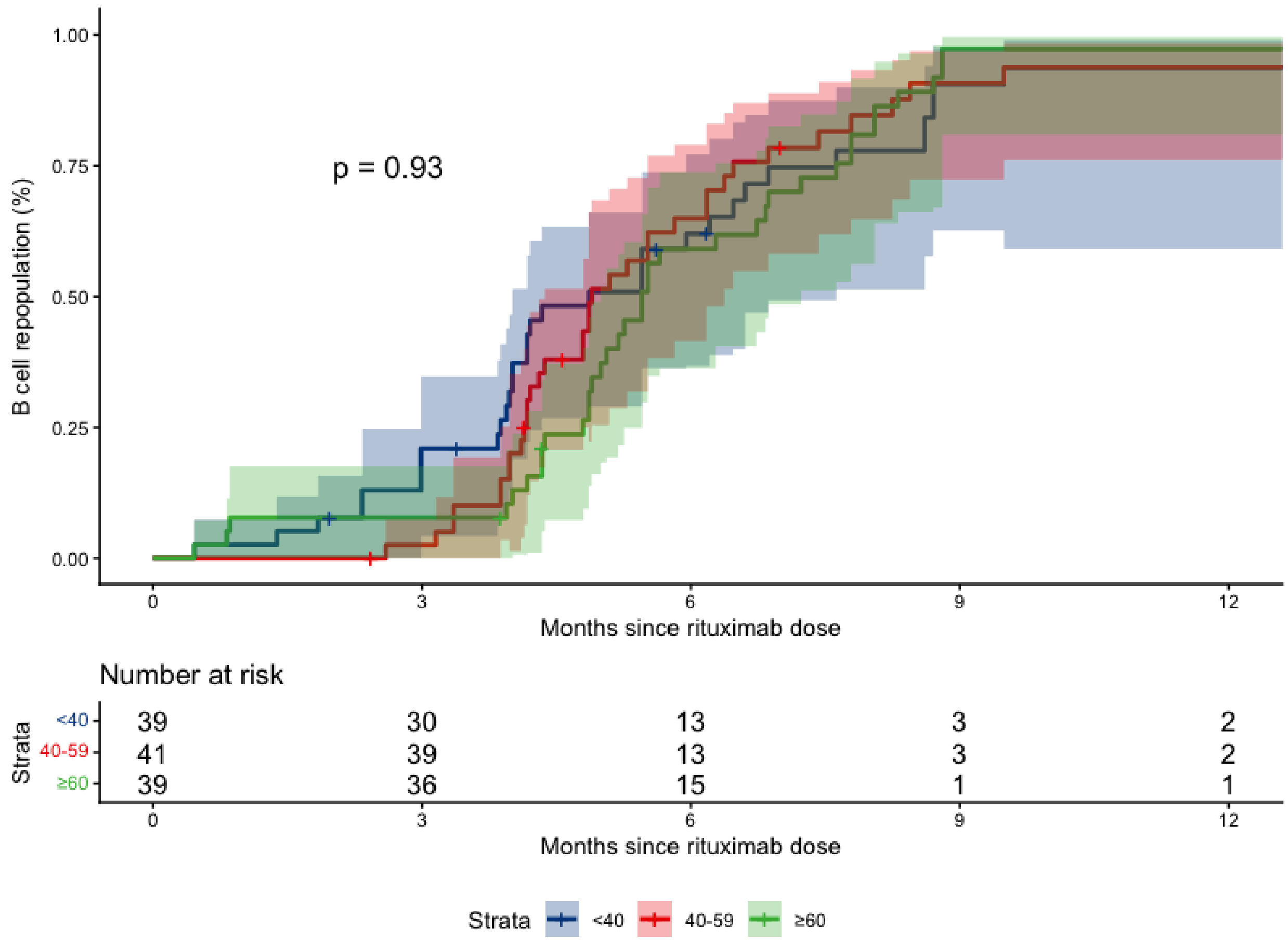

MPA: mycophenolic acid, AUC$_{0\text{-}12h}$: area under the concentration-time curve over 12 hours

Kaplan-Meier curve for time-to-B cell repopulation stratified by MPA-AUC (mg*hr/L) of <40 (blue), 40-59 (red), and ≥60 (green). The shaded area represents the confidence interval.

Supplemental Figure S2. Association between MPA-AUC$_{0\text{-}12h}$ and nephrotic syndrome relapse rate, among 119 mycophenolate observation windows in children that received prior rituximab treatment

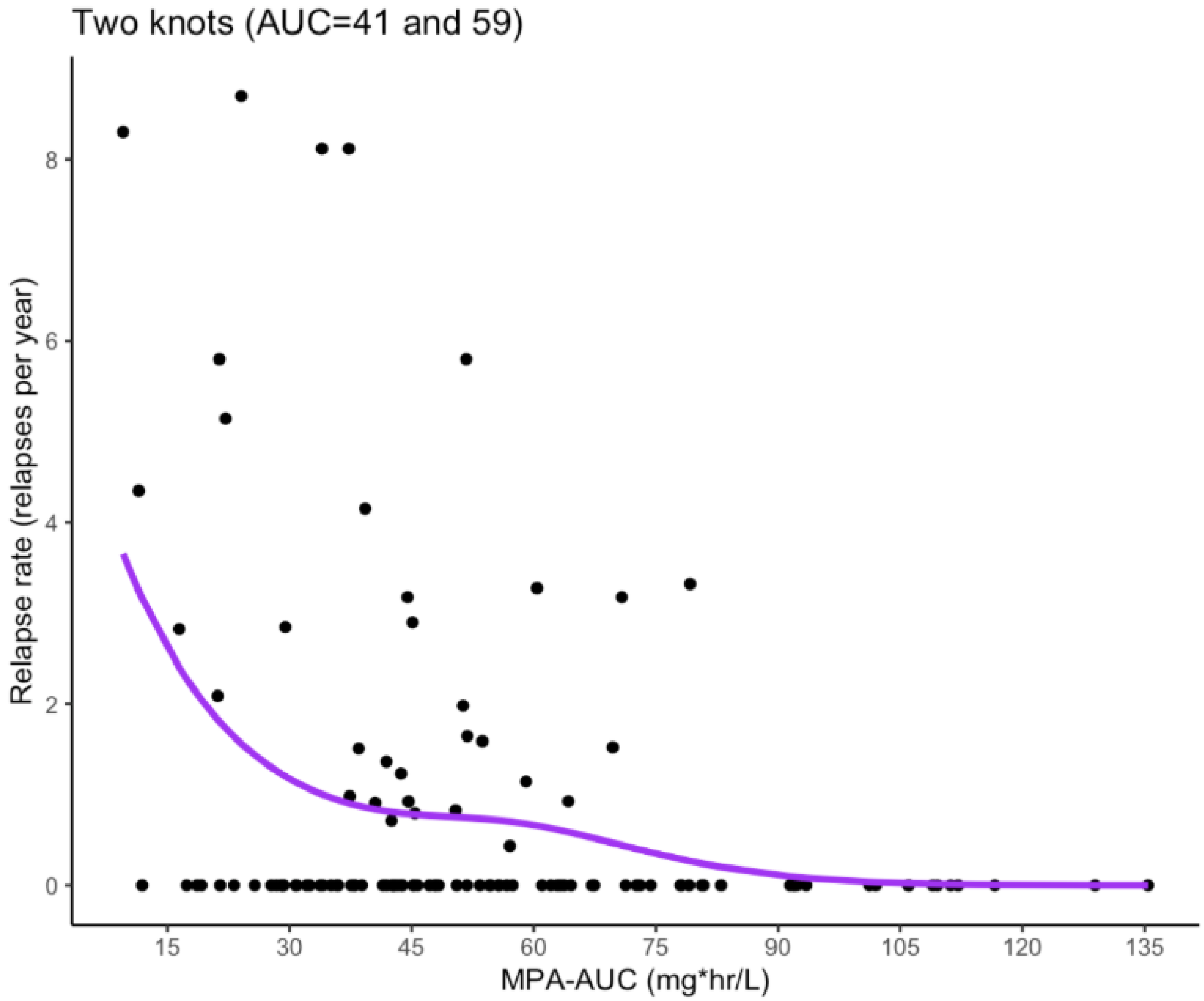

MPA: mycophenolic acid, AUC$_{0\text{-}12h}$: area under the concentration-time curve over 12 hours

Model adjusted for time since last rituximab treatment prior to the start of the current mycophenolate observation window.

Supplemental Figure S3. Youden index for optimal MPA-AUC$_{0\text{-}12h}$ threshold to predict a) relapse occurrence and b) neutropenia occurrence

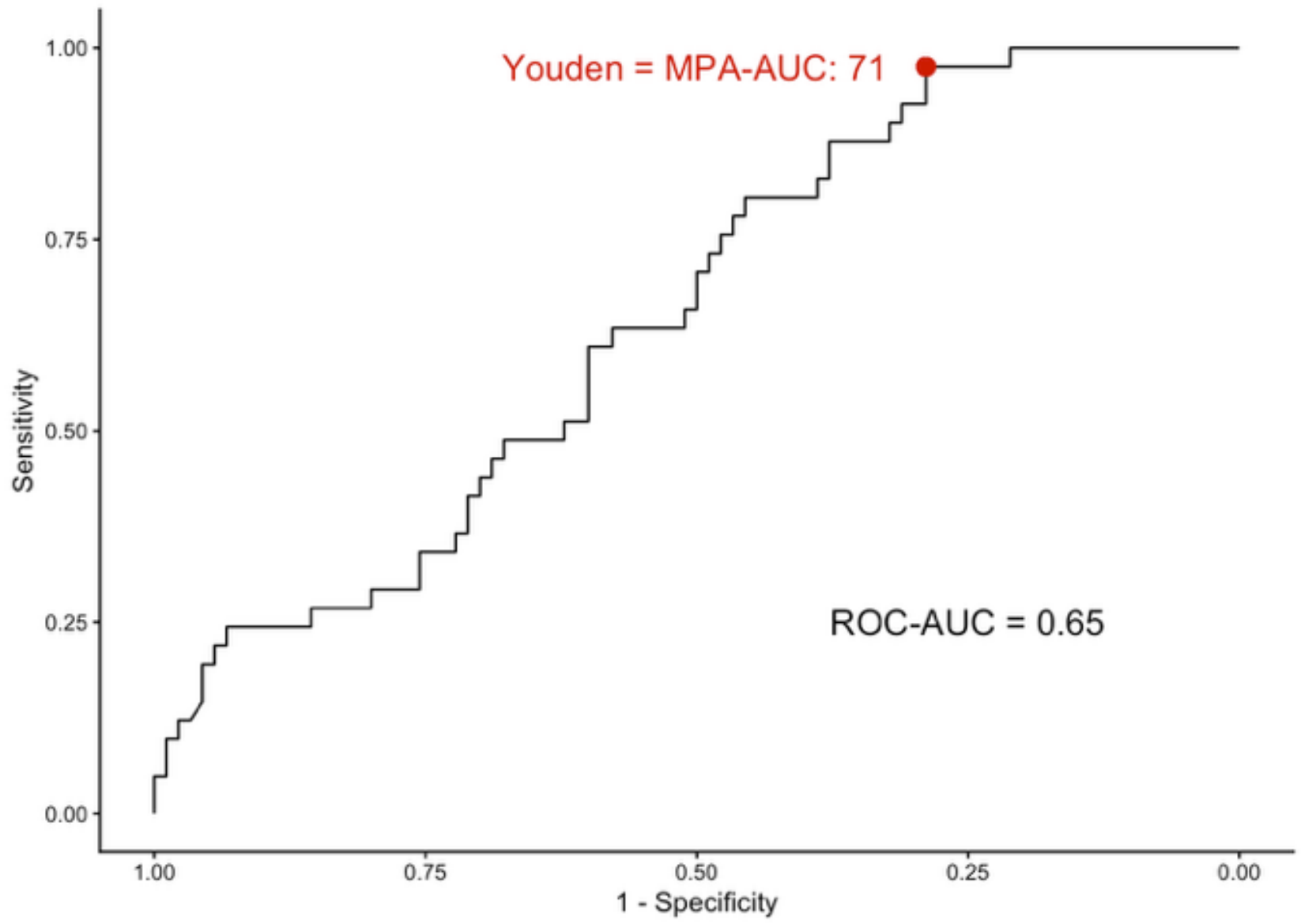

a)

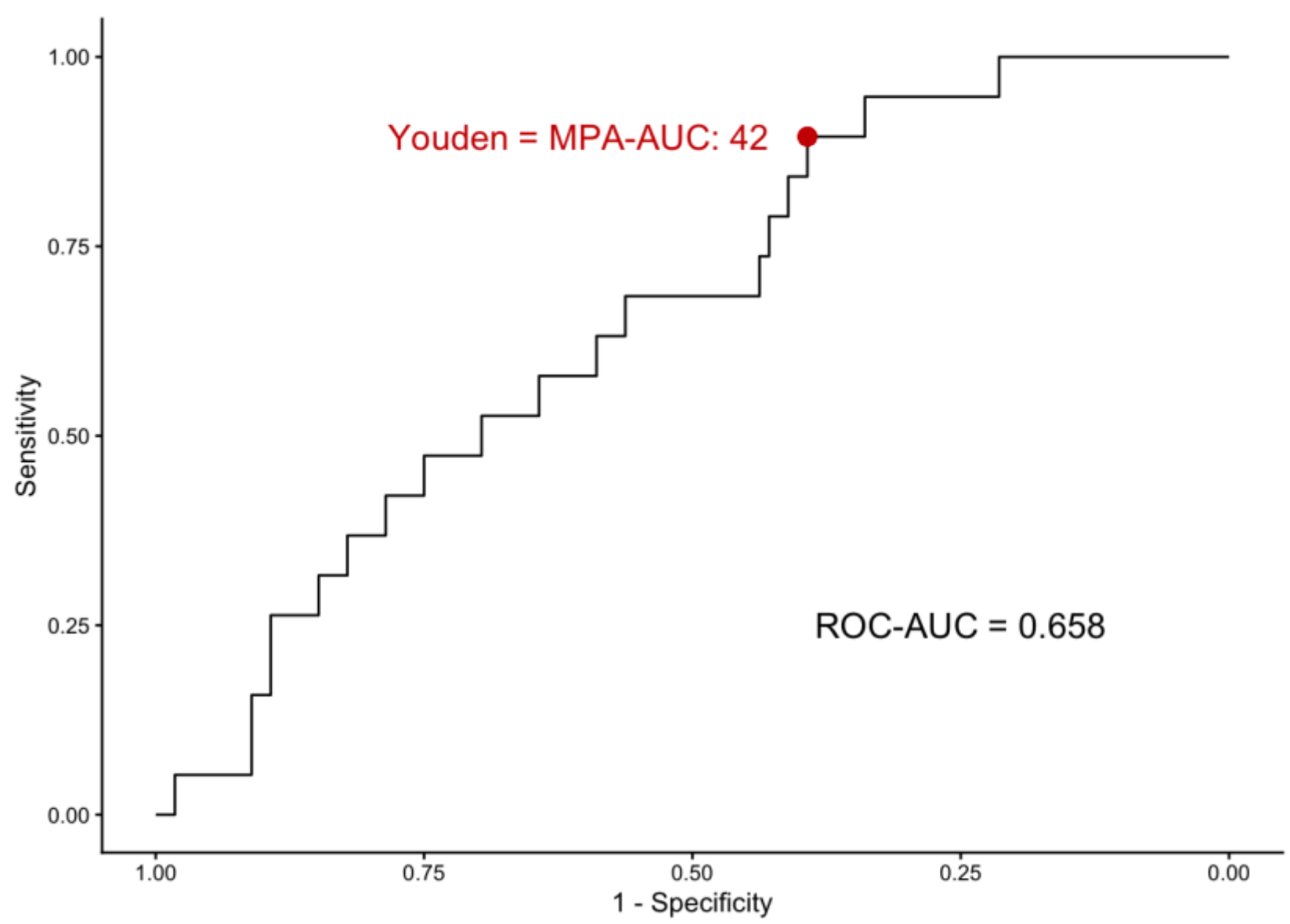

b)

a) Youden optimal MPA-AUC threshold to predict relapse occurrence during observation window = 71, Sensitivity = 98%, Specificity = 29%, **b**)Youden optimal MPA-AUC$_{0\text{-}12h}$ threshold to predict neutropenia occurrence during observation window = 42, Sensitivity = 90%, Specificity = 39%

STROBE Statement—Checklist of items that should be included in reports of ***cohort studies***

	Item No	Recommendation	Page No
**Title and abstract**	1	(*a*) Indicate the study's design with a commonly used term in the title or the abstract<br>(*b*) Provide in the abstract an informative and balanced summary of what was done and what was found	NA<br>2
**Introduction**			
Background/rationale	2	Explain the scientific background and rationale for the investigation being reported	4-5
Objectives	3	State specific objectives, including any prespecified hypotheses	5
**Methods**			
Study design	4	Present key elements of study design early in the paper	5-6
Setting	5	Describe the setting, locations, and relevant dates, including periods of recruitment, exposure, follow-up, and data collection	5-7
Participants	6	(*a*) Give the eligibility criteria, and the sources and methods of selection of participants. Describe methods of follow-up<br>(*b*) For matched studies, give matching criteria and number of exposed and unexposed	6-7
Variables	7	Clearly define all outcomes, exposures, predictors, potential confounders, and effect modifiers. Give diagnostic criteria, if applicable	8-10
Data sources/ measurement	8*	For each variable of interest, give sources of data and details of methods of assessment (measurement). Describe comparability of assessment methods if there is more than one group	Table 1, 4
Bias	9	Describe any efforts to address potential sources of bias	5-10
Study size	10	Explain how the study size was arrived at	6
Quantitative variables	11	Explain how quantitative variables were handled in the analyses. If applicable, describe which groupings were chosen and why	10-11, 14
Statistical methods	12	(*a*) Describe all statistical methods, including those used to control for confounding<br>(*b*) Describe any methods used to examine subgroups and interactions<br>(*c*) Explain how missing data were addressed<br>(*d*) If applicable, explain how loss to follow-up was addressed<br>(*e*) Describe any sensitivity analyses	9-11
**Results**			
Participants	13*	(a) Report numbers of individuals at each stage of study—eg numbers potentially eligible, examined for eligibility, confirmed eligible, included in the study, completing follow-up, and analysed<br>(b) Give reasons for non-participation at each stage<br>(c) Consider use of a flow diagram	Tab 1-4, Fig 1-4
Descriptive data	14*	(a) Give characteristics of study participants (eg demographic, clinical, social) and information on exposures and potential confounders<br>(b) Indicate number of participants with missing data for each variable of interest<br>(c) Summarise follow-up time (eg, average and total amount)	Table 1
Outcome data	15*	Report numbers of outcome events or summary measures over time	Fig 3/4

Main results	16	(*a*) Give unadjusted estimates and, if applicable, confounder-adjusted estimates and their precision (eg, 95% confidence interval). Make clear which confounders were adjusted for and why they were included<br>(*b*) Report category boundaries when continuous variables were categorized<br>(*c*) If relevant, consider translating estimates of relative risk into absolute risk for a meaningful time period	Table 2-4, Figure 1-4
Other analyses	17	Report other analyses done—eg analyses of subgroups and interactions, and sensitivity analyses	13, Supp Fig 1
**Discussion**			
Key results	18	Summarise key results with reference to study objectives	16
Limitations	19	Discuss limitations of the study, taking into account sources of potential bias or imprecision. Discuss both direction and magnitude of any potential bias	20
Interpretation	20	Give a cautious overall interpretation of results considering objectives, limitations, multiplicity of analyses, results from similar studies, and other relevant evidence	16-21
Generalisability	21	Discuss the generalisability (external validity) of the study results	20-21
**Other information**			
Funding	22	Give the source of funding and the role of the funders for the present study and, if applicable, for the original study on which the present article is based	22

*Give information separately for exposed and unexposed groups.

Note: An Explanation and Elaboration article discusses each checklist item and gives methodological background and published examples of transparent reporting. The STROBE checklist is best used in conjunction with this article (freely available on the Web sites of PLoS Medicine at http://www.plosmedicine.org/, Annals of Internal Medicine at http://www.annals.org/, and Epidemiology at http://www.epidem.com/). Information on the STROBE Initiative is available at http://www.strobe-statement.org.
